# Effect of Electron Blocking Layer Doping and Composition on the Performance of 310 nm Light Emitting Diodes

**DOI:** 10.3390/ma10121396

**Published:** 2017-12-06

**Authors:** Tim Kolbe, Arne Knauer, Jens Rass, Hyun Kyong Cho, Sylvia Hagedorn, Sven Einfeldt, Michael Kneissl, Markus Weyers

**Affiliations:** 1 Ferdinand-Braun-Institut, Leibniz-Institut für Höchstfrequenztechnik, Gustav-Kirchhoff-Str. 4, 12489 Berlin, Germany; Arne.Knauer@FBH-Berlin.de (A.K.); Jens.Rass@FBH-Berlin.de (J.R.); HyunKyong.Cho@FBH-Berlin.de (H.K.C.); Sylvia.Hagedorn@FBH-Berlin.de (S.H.); Sven.Einfeldt@fbh-berlin.de (S.E.); kneissl@physik.tu-berlin.de (M.K.); Markus.Weyers@FBH-Berlin.de (M.W.); 2 UVphotonics NT GmbH, Gustav-Kirchhoff-Str. 4, 12489 Berlin, Germany; 3 Institute of Solid State Physics, Technische Universität Berlin, Hardenbergstr. 36, 10623 Berlin, Germany

**Keywords:** light emitting diode, LED, ultraviolet, UV, electron blocking layer, EBL, MOVPE, doping, simulation, heterostructure

## Abstract

The effects of composition and p-doping profile of the AlGaN:Mg electron blocking layer (EBL) in 310 nm ultraviolet B (UV-B) light emitting diodes (LEDs) have been investigated. The carrier injection and internal quantum efficiency of the LEDs were simulated and compared to electroluminescence measurements. The light output power depends strongly on the temporal biscyclopentadienylmagnesium (Cp${}_{2}$Mg) carrier gas flow profile during growth as well as on the aluminum profile of the AlGaN:Mg EBL. The highest emission power has been found for an EBL with the highest Cp${}_{2}$Mg carrier gas flow and a gradually decreasing aluminum content in direction to the p-side of the LED. This effect is attributed to an improved carrier injection and confinement that prevents electron leakage into the p-doped region of the LED with a simultaneously enhanced carrier injection into the active region.

## 1. Introduction

(In)AlGaN based LEDs in the UV-B spectral range are promising candidates that could replace established UV light sources in various applications, e.g., medical diagnostics, phototherapy, and plant growth lighting [1,2]. However, despite the enormous progress that UV-B LEDs have made, the performance characteristics of these devices are still suffering from high defect densities and poor carrier injection compared to visible LEDs [3,4]. Today, the best UV-B LEDs exhibit external quantum efficiencies of only a few percent [5,6].

The optimization of the LED heterostructure, especially of the quantum well active region and the EBL, is one way to increase the efficiency of UV-B LEDs. One promising approach to improve the efficiency is to increase the radiative recombination rates in the active region. Therefore, we have previously discussed the influence of the quantum-well (QW) and quantum-well-barrier (QWB) composition [7], the number of QWs [8] and the QW width [9] on the emission characteristics of UV-B LEDs. The second key challenge to realize efficient UV-B LEDs is an efficient carrier injection. This is mainly realized by a magnesium (Mg) doped AlGaN EBL that prevents electron leakage into the p-doped top layers without impairing efficient hole injection into the active region of the LED. In previous studies, mostly Al${}_{x}$Ga${}_{1-x}$N:Mg EBLs with constant aluminum mole fraction *x* and constant p-doping over the whole EBL have been reported for LEDs in the UV-B spectral range [10,11,12,13,14,15]. Only some theoretical studies regarding different EBL designs have been published [16,17,18,19,20]. In addition to the aluminum content *x*, the thickness and the p-doping is also critical for the functionality of the Al${}_{x}$Ga${}_{1-x}$N:Mg EBL. Tu et al. [21] have shown that the efficiency decreases for very high or very low magnesium concentrations in the EBL. However, systematic investigations of EBL designs have not yet been presented for UV-B LEDs.

In this paper, we report on the effects of different temporal Cp${}_{2}$Mg carrier gas flow profiles during growth of the AlGaN:Mg EBL and different compositional designs of the EBL on the emission characteristics of 310 nm LEDs. The results of electroluminescence measurements will be discussed in combination with simulations of the carrier injection into the (In)AlGaN multiple quantum well active region of the UV-B LEDs.

## 2. Experimental

**Figure 1:**
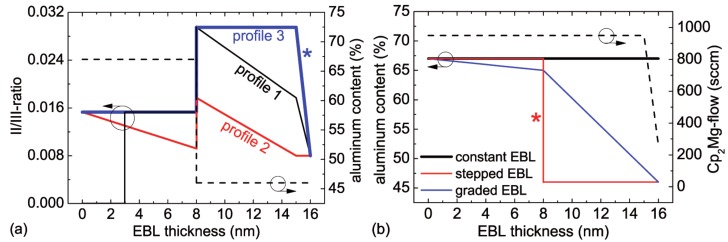
Illustration of the different EBL designs: (**a**) temporal Cp${}_{2}$Mg carrier gas flow profiles (sample series 1) and (**b**) aluminum composition profiles (sample series 2). The curves in (**a**,**b**) corresponding to the same EBL design are marked by a star.

The LED heterostructures were grown by metal organic vapor phase epitaxy (MOVPE) on (0001) oriented sapphire substrates. After the deposition of 1500 nm AlN at elevated temperature, a 200 nm AlN/GaN short-period superlattice was grown followed by a 500 nm undoped and 4.5 μm silicon doped Al${}_{0.5}$Ga${}_{0.5}$N contact layer, a threefold (In)AlGaN/(In)AlGaN multiple quantum well active region, a 16 nm thick Mg-doped EBL, a 140 nm thick Al${}_{0.42}$Ga${}_{0.58}$N:Mg/Al${}_{0.32}$Ga${}_{0.68}$N:Mg short-period superlattice (SL) and a 20 nm GaN:Mg contact layer. The AlN base layer on sapphire shows a typical full width at half maximum of X-ray rocking curves of 80 arcsec for the (0002) reflection and 900 arcsec for the (30$\bar{3}$2) reflection. For p-type activation, the samples were annealed in-situ at 890 ${}^{\circ }$C for 15 min in a nitrogen ambience. In a first series of samples, the influence of the temporal Cp${}_{2}$Mg carrier gas flow profile (flow of H${}_{2}$ carrier gas through the Cp${}_{2}$Mg bubbler at a temperature of 18 ${}^{\circ }$C and a pressure of 600 Torr) during growth of the EBL on the emission characteristics of the LEDs was investigated. Therefore, the Cp${}_{2}$Mg carrier gas flow during the growth of a stepped 8 nm Al${}_{0.67}$Ga${}_{0.33}$N:Mg/8 nm Al${}_{0.46}$Ga${}_{0.54}$N:Mg EBL was varied. In flow profile 1, the first 3 nm of the EBL were undoped (Cp${}_{2}$Mg carrier gas flow zero), the next 5 nm were doped with a flow of 950 sccm followed by a reduction from 950 sccm to 570 sccm over 7 nm and a further reduction from 570 sccm to 259 sccm over 1 nm. The EBL from the LED with the flow profile 2 had the following sequence of Cp${}_{2}$Mg carrier gas flows: the first 8 nm was doped with a flow gradually decreasing from 950 sccm to 570 sccm, followed by a further reduction from 570 sccm to 259 sccm over the following 8 nm. The EBL with flow profile 3 was grown with a constant flow of 950 sccm over 15 nm followed by a reduction to 259 sccm over 1 nm. The corresponding changes of the II/III-ratio are illustrated in Figure 1a. In a second series of samples, the aluminum composition profile of the EBL was varied. We assume that the composition profile of the solid follows linearly the temporal course of the composition of the vapor phase during the growth. Thereby, LEDs with constant aluminum mole fraction in the EBL (16 nm Al${}_{0.67}$Ga${}_{0.33}$N:Mg EBL), a stepped EBL (8 nm Al${}_{0.67}$Ga${}_{0.33}$N:Mg/8 nm Al${}_{0.46}$Ga${}_{0.54}$N:Mg) and a graded EBL with a gradually decreasing aluminum content in growth direction (8 nm Al${}_{0.67}$Ga${}_{0.33}$N:Mg to Al${}_{0.65}$Ga${}_{0.35}$N:Mg/8 nm Al${}_{0.65}$Ga${}_{0.35}$N:Mg to Al${}_{0.46}$Ga${}_{0.54}$N:Mg) were compared. The Cp${}_{2}$Mg carrier gas flow during the growth of the EBL was kept constant for this series. For a better illustration, the variants are illustrated in Figure 1b. The composition of the EBL was determined by high-resolution X-ray diffraction measurements (Philips X’Pert Pro MRD, PANalytical B.V., Almelo, The Netherlands) and secondary ion mass spectrometry (RTGMikroanalyse GmbH, Berlin, Germany) on calibration samples.

LEDs were fabricated using standard chip-processing technologies. Mesa structures with a p-contact area of about 0.15 mm${}^{2}$ were defined by inductively-coupled plasma etching in order to expose the n-AlGaN surface. Platinum-based p-contacts and vanadium-based n-contacts were deposited to form the p-electrode and the n-electrode, respectively.

The electrical and optical characteristics of the LEDs were measured on-wafer under direct current (dc) injection. For that purpose, the wafers were placed epi-side up on a sample holder without any active cooling. The emission spectra and the optical power vs. current (L-I) characteristics were measured by collecting the light emitted through the substrate with an optical fiber spectrometer (Ocean Optics USB4000, Ocean Optics, Inc., Largo, LA, USA) and a calibrated silicon photodiode (Hamamatsu S2281-01, Hamamatsu Photonics, Hamamatsu, Japan), respectively. To explain the experimental data, the carrier injection into the active region has been simulated based on a one-dimensional drift-diffusion model [22]. A nonradiative carrier lifetime of 9 ns, an electron mobility of 100 cm${}^{2}$ V${}^{-1}$ s${}^{-1}$, a hole mobility of 5 cm${}^{2}$ V${}^{-1}$ s${}^{-1}$, a donor concentration of 5 × 10${}^{18}$ cm${}^{-3}$ with a donor ionization energy of 13 meV and an acceptor concentration of 6 × 10${}^{19}$ cm${}^{-3}$ (Cp${}_{2}$Mg carrier gas flow: 950 sccm), 1 × 10${}^{19}$ cm${}^{-3}$ (570 sccm) and 5 × 10${}^{18}$ cm${}^{-3}$ (259 sccm) for the AlGaN:Mg EBL and 2 × 10${}^{19}$ cm${}^{-3}$ for the (Al)GaN:Mg layers with an acceptor ionization energy of 170 meV for GaN and 510 meV for AlN (linear interpolation between these values for AlGaN) were assumed.

## 3. Results and Discussion

### 3.1. Temporal Cp${}_{2}$Mg Carrier Gas Flow Profile

**Figure 2:**
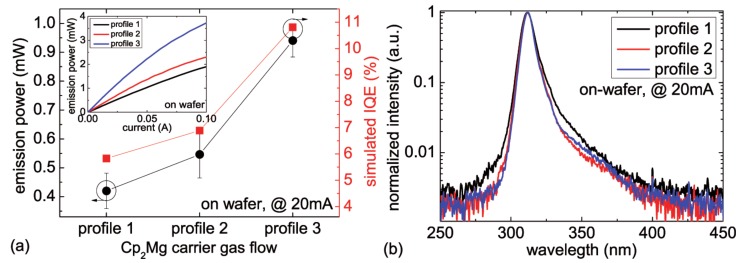
(**a**) measured average emission power (at 20 mA, on-wafer) as well as simulated internal quantum efficiency (IQE) and (**b**) typical normalized emission spectra (at 20 mA, on-wafer) of 310 nm LEDs with different temporal Cp${}_{2}$Mg carrier gas flow profiles in the EBL. Inset in (**a**): typical emission power vs. current characteristics of 310 nm LEDs with different temporal Cp${}_{2}$Mg carrier gas flow profiles of the EBL.

In this series, the aluminum composition profile of the EBL was kept constant (stepped EBL) and the Cp${}_{2}$Mg carrier gas flow was varied during the growth of the EBL. Figure 2a shows the averaged (>250 LEDs per wafer) on-wafer emission power of 310 nm LEDs with the temporal Cp${}_{2}$Mg carrier gas flow profiles 1, 2 and 3 measured at 20 mA. Typical emission power vs. current characteristics of the LEDs are shown in the inset of Figure 2a.

The highest emission power can be found for LEDs with profile 3, which corresponds to the most heavily doped EBL. The LEDs with the decreasing magnesium supply in growth direction (profile 2) show around 40% lower emission power. The lowest emission power is observed for the LEDs with profile 1. Here, the first 3 nm of the EBL were undoped followed by a nominally higher p-doping level compared to profile 2. Typical normalized emission spectra of the different LEDs recorded at 20 mA are shown in Figure 2b. All LEDs exhibit single peak emission at 310 nm, which indicates low electron leakage to the p-side.

To explain the experimental data, the carrier injection into the active region has been simulated based on a one-dimensional drift-diffusion model [22]. Figure 2a also shows the simulated internal quantum efficiency (IQE) of the investigated LED structures. The trend of the simulated data is in good agreement to the experimental data. Therefore, the device simulations are assumed to be appropriate to analyse the differences in the carrier distribution between the investigated LEDs.

**Figure 3:**
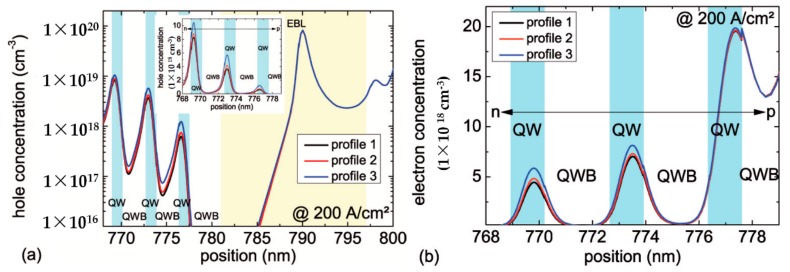
Simulated (**a**) hole concentration and (**b**) electron concentration of 310 nm LEDs with different doping profiles in the EBL (at 200 A/cm${}^{2}$).

The calculated hole and electron concentrations in the active region at a current density of 200 A/cm${}^{2}$ are shown in Figure 3a,b, respectively. The hole and electron concentration and therefore the radiative recombination rate are highest for the LED structure with profile 3. This can be attributed to the highest magnesium concentration and thus the highest hole concentration in this LED in comparison to the other two. Furthermore, the simulations of the three structures show different energetic shifts of the valence and conduction band edge of the EBL, which influence the effective band offset between the last quantum well barrier and the EBL (not shown here). Electrons in the LED structure with profile 3 see a 15.5 meV and 20.3 meV higher potential barrier for the electrons compared to electrons in the LED structures with profile 2 and 1, respectively. Therefore, the electron leakage current is reduced with increasing magnesium doping, which results in a higher electron concentration in the active region. The lowest carrier concentration in the active region is observed for the LED structure with profile 1. In this case, the first undoped 3 nm of the EBL results in an inefficient hole injection into the active region and an elevated electron leakage over the EBL because of the reduced EBL potential barrier height.

[-15]For a further enhancement of the carrier injection into the active region, other doping profiles or an even further increase of the p-doping level of the EBL could be a worthwile approach. However, it should be considered that at a too high p-doping level self-compensating effects [23,24] take place, which reduce the device performance. Finally, it should be noted that a variation of other parameters like the MOVPE reactor geometry as well as growth conditions (temperature, reactor pressure, etc.) may shift the optimum Cp${}_{2}$Mg carrier gas flow to a value, which is different from the one reported here.

### 3.2. Aluminum Composition Profile

**Figure 4:**
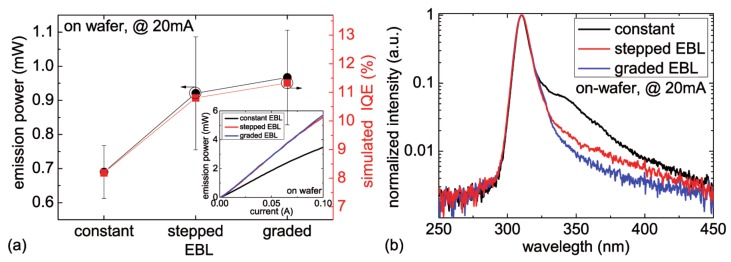
(**a**) measured average emission power (at 20 mA, on-wafer) as well as simulated IQE and (**b**) typical normalized emission spectra (at 20 mA, on-wafer) of 310 nm LEDs with different profiles of the aluminum composition in the EBL. Inset in (**a**): typical emission power vs. current characteristics of 310 nm LEDs with these different EBL designs.

Another key parameter to realize an efficient EBL is its aluminum composition profile. LEDs with a constant, a stepped and a graded aluminum composition are compared. The Cp${}_{2}$Mg carrier gas flow during the growth of all samples was kept constant. Figure 4a shows the averaged (>250 LEDs per wafer) on-wafer emission power of these 310 nm LEDs with different EBL designs measured at 20 mA. The inset of Figure 4a represents a typical emission power vs. current characteristics for each type of LED. The highest emission power is found for the LEDs with the graded EBL, closely followed by the LEDs with the stepped EBL. The LEDs with the constant aluminum content in the EBL show around 30% lower emission power compared to the other two LED types. Figure 4b shows typical normalized emission spectra of these LEDs operated at 20 mA. For all LEDs, the emission spectra are dominated by the multi-quantum-well emission at 310 nm. The luminescence near 350 nm, which is especially visible for the LED with the constant EBL, can be attributed to carrier recombination in the p-type AlGaN/AlGaN short-period superlattice [25] due to electron leakage.

Figure 4a also shows the simulated IQE of the investigated LED structures. Once again, there is a good agreement of the trend of the experimental and simulated data.

**Figure 5:**
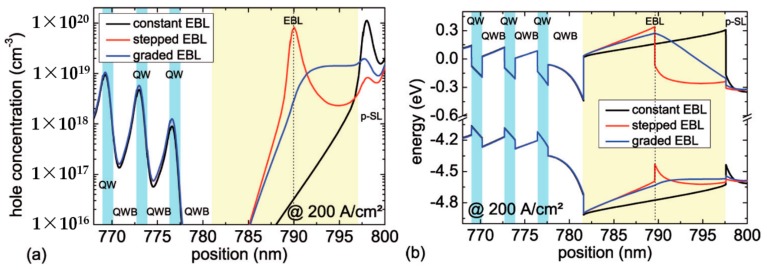
Simulated (**a**) hole concentration and (**b**) conduction- and valence band edge of 310 nm LEDs with different EBL designs (at 200 A/cm${}^{2}$).

The calculated hole concentrations as well as the calculated conduction and valence band profiles around the active region and the EBL at a current density of 200 A/cm${}^{2}$ are shown in Figure 5a,b, respectively. The data indicates that the highest hole and electron concentration (electron concentration is not shown here) and, therefore, the highest radiative recombination rate can be found for the LED structure with the graded EBL. A slightly lower carrier concentration can be observed for the LED structure with the stepped EBL. The carrier concentration is lowest for the LED with the constant EBL. This can be explained by the band structure around the EBL. On the one hand, the EBL of the LEDs with the graded and stepped EBL results in a higher potential barrier in the conduction band for the electrons in comparison to the constant EBL. Therefore, the electron leakage current is reduced (see also low parasitic luminescence in Figure 4b) and the electron concentration in the active region is enhanced. On the other hand, the LEDs with the graded and stepped EBL show only a 28 meV high potential barrier for the holes in the valence band at the upper EBL interface. In contrast, this potential barrier is around 189 meV for the LED with the constant EBL which leads to an accumulation of holes and a reduced hole injection into the active region. An analysis of the simulation results suggests that the accumulation of holes in the middle of the stepped EBL does not considerably hinder the hole injection. Therefore, the combination of the improved electron confinement in the active region and the improved hole injection into the active region results in an enhancement of the emission power of LEDs with the stepped and graded EBL design compared to the LEDs with the commonly used constant EBL design.

## 4. Conclusions

The effect of the magnesium doping profile and the aluminum composition profile in the EBL on the performance of 310 nm LEDs was investigated by electroluminescence measurements and numerical simulations of the band structure and the carrier concentration. The highest emission power was found experimentally and theoretically for LEDs with the highest p-doping level in the EBL and an EBL whose aluminum content gradually decreases in growth direction. This effect is attributed to an improved carrier injection into the active region and an improved confinement that prevents electron leakage into the p-doped region of the LED.

## Abbreviations

The following abbreviations are used in this manuscript:

II/III-ratio	group II to group III ratio
AlGaN	aluminum gallium nitride
AlN	aluminum nitride
Cp${}_{2}$Mg	biscyclopentadienylmagnesium
EBL	electron blocking layer
H	hydrogen
InAlGaN	indium aluminum gallium nitride
IQE	internal quantum efficiency
LED	light emitting diode
Mg	magnesium
MOVPE	metal organic vapor phase epitaxy
QW	quantum well
QWB	quantum well barrier
SL	super lattice
UV	ultraviolet
UV-B	ultraviolet B
